# Selective intracellular vaporisation of antibody-conjugated phase-change nano-droplets *in vitro*

**DOI:** 10.1038/srep44077

**Published:** 2017-03-23

**Authors:** A. Ishijima, K. Minamihata, S. Yamaguchi, S. Yamahira, R. Ichikawa, E. Kobayashi, M. Iijima, Y. Shibasaki, T. Azuma, T. Nagamune, I. Sakuma

**Affiliations:** 1 Department of Precision Engineering, School of Engineering, The University of Tokyo, Tokyo 113-8656, Japan; 2 Department of Applied Chemistry, Kyushu University, Fukuoka 819-0395, Japan; 3 Department of Chemistry & Biotechnology, School of Engineering, The University of Tokyo, Tokyo 113-8656, Japan; 4 Research Center for Advanced Science and Technology, The University of Tokyo, Tokyo 153-8904, Japan; 5 Center for Disease Biology and Integrative Medicine, The University of Tokyo, Tokyo 113-8656, Japan; 6 Department of Bioengineering, The University of Tokyo, Tokyo 113-8656, Japan; 7 Medical Device Development and Regulation Research Center, School of Engineering, The University of Tokyo, Tokyo 113-8656, Japan

## Abstract

While chemotherapy is a major mode of cancer therapeutics, its efficacy is limited by systemic toxicities and drug resistance. Recent advances in nanomedicine provide the opportunity to reduce systemic toxicities. However, drug resistance remains a major challenge in cancer treatment research. Here we developed a nanomedicine composed of a phase-change nano-droplet (PCND) and an anti-cancer antibody (9E5), proposing the concept of ultrasound cancer therapy with intracellular vaporisation. PCND is a liquid perfluorocarbon nanoparticle with a liquid–gas phase that is transformable upon exposure to ultrasound. 9E5 is a monoclonal antibody targeting epiregulin (EREG). We found that 9E5-conjugated PCNDs are selectively internalised into targeted cancer cells and kill the cells dynamically by ultrasound-induced intracellular vaporisation. *In vitro* experiments show that 9E5-conjugated PCND targets 97.8% of high-EREG-expressing cancer cells and kills 57% of those targeted upon exposure to ultrasound. Furthermore, direct observation of the intracellular vaporisation process revealed the significant morphological alterations of cells and the release of intracellular contents.

Tremendous efforts have been devoted to curing cancer^1^,^2^,^3^. Surgery, radiotherapy and chemotherapy are the current major modes of cancer treatment^4^,^5^,^6^. Visually non-detectable, very early-stage, invasive, metastatic and boundary indistinct cancer are difficult to treat with surgery or radiotherapy^7^,^8^. Furthermore, such treatments require a boundary on the order of millimetres between the targeted area and the conservation area to account for the accuracy of surgical instruments or particle beams. Chemotherapy is frequently combined with these physical treatment modes to overcome such limitations^9^,^10^. However, systemic toxicities and limited treatment efficacy caused by drug resistance limit the success of chemotherapy^11^,^12^.

Recent advances in targeted drug delivery using nanotechnology allow for a breakthrough in chemotherapy^13^,^14^. General cancer nanomedicine is the local/selective delivery of carriers loaded with anti-cancer drugs to cancerous tissue sites and the exhibition of a treatment upon reaching the target^15^,^16^. These are prepared using materials such as polymers, lipids and viruses^13^,^17^ and small enough (from nanometre down to sub-micrometre scale) to be passively transported to extravascular cancer sites through enhanced permeability and retention (EPR) effects: the large endothelial cell gap junctions of tumour blood vessels (~200−1200 nm) permit leakage of large particles into the interstitial space, severely impairing lymphatic drainage^18^,^19^,^20^,^21^. Bioconjugation of antibodies to nanomedicines further improves their specificity to target cancer cells and potentially induces receptor-mediated endocytosis for their intracellular delivery^13^,^22^,^23^. However, the drug-resistance properties of cancer, especially drug efflux pumps, are of great concern in cancer nanomedicine because the treatment involves the release of loaded chemical drugs^17^,^24^,^25^.

Here we developed an ultrasound-activated nanomedicine for cancer-targeted ultrasound therapy that physically treats cancer cells. We proposed a new platform of cancer therapy that comprises ultrasound, antibodies and ultrasound-triggered particles. Ultrasound-triggering provides the benefits of non-invasiveness, deep penetration (more than cm-order) and sub-millimetre to millimetre-order spatial controlling capability of ultrasound-beam-focusing that enables high spatial-temporal control of therapeutic activation. Active targeting is a potential approach to achieve intracellular delivery of the nanomedicine. An antibody possessing strong and specific antigen recognition ability often induces endocytosis upon binding to the antigen expressed on the surface of cancer cells^22^,^23^. Epiregulin (EREG), the cell-membrane-expressed ligand of epidermal growth factor receptor, is expressed and integrated into the plasma membrane at relatively high levels in a variety of human cancers, including colorectal and breast cancer^26^. This ligand has been intensively investigated as a therapeutic target^26^. The anti-EREG antibody 9E5 was conjugated as the active targeting moiety to submicron particles called phase-change nano-droplets (PCNDs), acoustic droplets composed of a phospholipid shells and liquid perfluorocarbon (PFC) core (Fig. 1a). These nano-sized PFC droplets have attracted attention as multi-modal imaging contrast agents and drug carriers^27^,^28^,^29^,^30^ because they vaporise into microbubbles upon exposure to ultrasound^31^. We attempted to utilise this feature to physically kill cancer cells by intracellular vaporisation. Once 9E5-conjugated PCNDs were internalised to cells, ultrasound exposure vaporises PCNDs and those liquid-to-gas transition phenomena is considered to induce significant damage to cells (Fig. 1b). Here, we succeeded in demonstrating the selective targeting and cytotoxic effects *in vitro* with direct observation of intracellular vaporisation by high-speed imaging.

## Results

### Synthesis of 9E5-conjugated PCND

**Figure 1:**
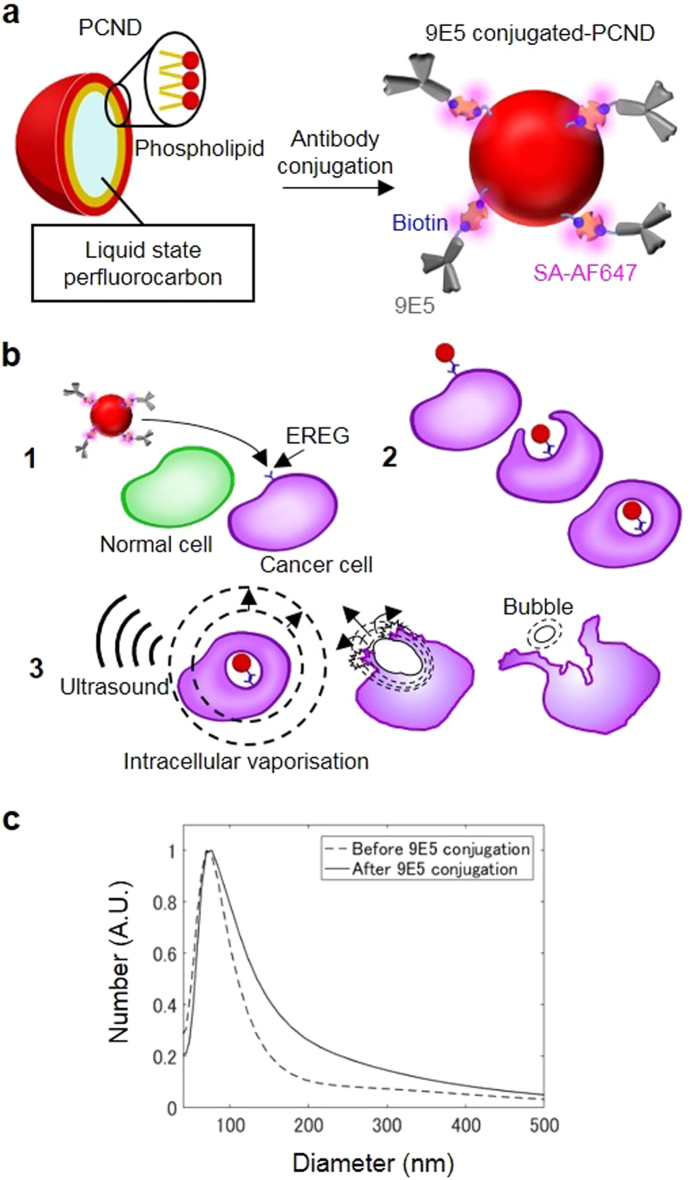
Schematic diagrams for explaining the concept of intracellular vaporisation cancer therapy and size distribution of 9E5-conjugated PCND. (**a**) Illustration of 9E5-conjugated PCND. (**b**) Schematic diagrams of selective intracellular vaporisation in cancer cells.9E5-conjugated PCND selectively internalised inside cancer cells via 9E5-mediated endocytosis (**1**–**2**), and vaporisation by ultrasound exposure killed these cells (**3**). (**c**) Size distributions of PCNDs before and after 9E5 conjugation. Size distributions before (dashed line) and after conjugation (solid line) were measured using a laser diffraction particle analyser.

9E5-conjugated PCNDs consists of a PFC liquid core (a mixture of perfluoropentane and perfluorohexane), a phospholipid shell and antibody 9E5. The 9E5 human anti-EREG antibody was selected for active targeting of PCNDs. In a preliminary experiment, fluorescent-labelled 9E5 antibody clearly bound to high-EREG-expressing cells, followed by rapid internalisation into intracellular compartments within a few hours (Supplementary Fig. 1). 9E5 was conjugated to PCNDs using the biotin-streptavidin-biotin binding technique (see Methods). The 9E5 was purified from the peritoneal fluid of mice transplanted with hybridoma cells secreting 9E5. Biotinylated-9E5 and Alexa Fluor 647-conjugated streptavidin (SA-AF647) were bound to biotinylated PCND. The mean diameter of 9E5-conjugated PCNDs was 140 ± 120 nm (Fig. 1c).

### Targeting capability and 9E5-conjugated PCND internalisation

**Figure 2:**
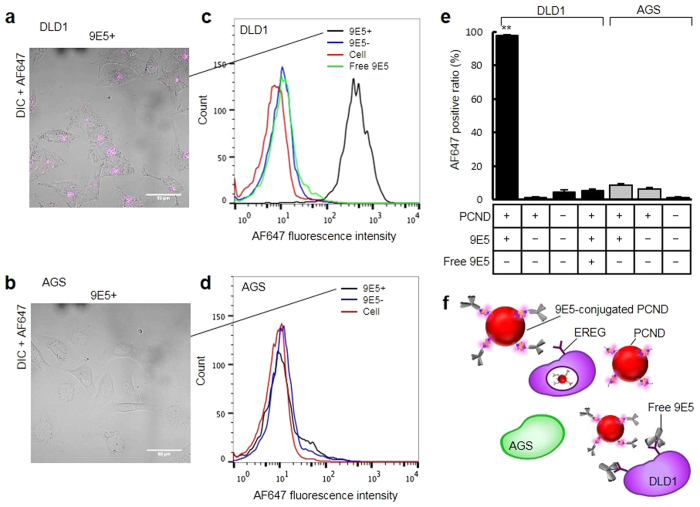
Selective internalisation of 9E5-conjugated PCND into high-EREG-expressing cells. (**a**,**b**) Confocal microscopic observation of selective internalisation of AF647-labelled 9E5-conjugated PCNDs. The merged image of the pink fluorescent (AF647 on 9E5-conjugated PCNDs) and differential interference contrast (DIC) images was obtained from DLD1 and AGS cells. Scale bars were 50 μm. (**c**–**e**) Flow cytometry analysis of cells treated with AF647-labelled 9E5-conjugated PCNDs. Histograms of the AF647 fluorescence intensity were obtained by analysing the treated DLD1 (**c**) and AGS cells (**d**). Cells were treated with AF647-labelled 9E5-conjugated PCNDs (black line), AF647-labelled non-conjugated PCNDs (blue line) and both AF647-labelled 9E5-conjugated PCNDs and excess free 9E5 (green line) as well as not treated (red line). (**e**) The AF647-positive ratio was determined as the ratio of the AF647-fluorescence-positive cells from the histograms. Each bars represent the mean ± S.D. (*N* = 5). **Indicates *p* < 0.01 by one-way ANOVA followed by the Tukey method. (**f**) Schematic illustrations of selective internalisation into DLD1 cells and competitive blocking of internalisation with free 9E5.

Conjugation of 9E5 to PCNDs permits the selective targeting of PCNDs to high-EREG-expressing cells and induces internalisation by endocytosis. The human colonic adenocarcinoma cell line DLD1 and the human gastric cancer cell line AGS were selected as high and low EREG-expressing cancer cell lines, respectively. To demonstrate the selective targeting capability of 9E5-conjugated PCNDs to DLD1 cells, we used SA-AF647 as a probe. The targeted cells were observed by confocal laser scanning microscopy (CLSM), and the number of PCNDs attached and the fraction of bound DLD1 cells were quantitatively measured by flow cytometer. Figure 2a,b shows CLSM observation of antibody-mediated accumulation and internalisation of AF647-labelled 9E5-conjugated PCNDs to DLD1 and AGS cells. The pink fluorescent signals from AF647 were only observed from DLD1 cells (Supplementary Fig. 2, Iijima *et al*. in preparation). To further investigate the location of 9E5-conjugated PCNDs, cell membranes were stained with DiO dye, which exhibits green fluorescence. As shown in Supplementary Fig. 3, cell membranes labelled with green and pink fluorescent signals from AF647 were observed inside DLD1 cells (Supplementary Movies 1 and 2). These CLSM images clearly show the internalisation of 9E5-conjugated PCNDs into DLD1 cells, whereas no clear fluorescence signals were observed from the other types of cells.

Figure 2c–e shows the results of flow cytometry analysis to evaluate the targeting capabilities of 9E5-conjugated PCNDs toward DLD1 cells (*N *= 5). Results indicate that the 97.8 ± 0.5% of DLD1 cells were targeted by 9E5-conjugated PCNDs, whereas 1.4 ± 0.3% of DLD1 cells were targeted by non-9E5-conjugated PCNDs, similar to that of the control (4.4 ± 1.6%). Furthermore, the ratio of AGS cells targeted by 9E5-conjugated (8.7 ± 1.0%) and non-9E5-conjugated PCNDs (6.5 ± 1.0%) were close to the levels of the control. Pre-treatment of DLD1 cells with free 9E5 and co-treatment with 9E5-conjugated PCNDs significantly decreased the targeting efficiency of 9E5-conjugated PCNDs (5.4 ± 0.9%), indicating that recognition of EREG by 9E5 antibody plays an important role in the targeting of PCNDs to DLD1 cells (Fig. 2f).

These results show that 9E5-conjugated PCNDs selectively targeted and were internalised by DLD1 cells (high EREG expression) but not AGS cells (low EREG expression). Moreover, PCNDs non-conjugated to 9E5 antibody displayed no targeting capability toward DLD1 cells.

### Intracellular vaporisation of 9E5-conjugated PCND

**Figure 3:**
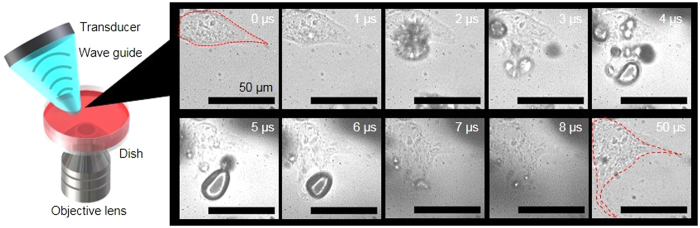
High-speed imaging of intracellular vaporisation. High-speed images were recorded at 1 Mfps with 101 subsequent frames to monitor the intracellular vaporisation processes of 9E5-conjugated PCND in DLD1 cells and partially represented at the interval of 1μs from 0 to 8 μs and additionally at 50 μs. The red dashed lines in the images at 0 and 50 μs represent the outline of cells before and after vaporisation. Scale bars were 50 μm (See also Supplementary Movies 3 and 4).

We showed that the 9E5-conjugated PCNDs accumulate selectively inside DLD1 cells. Next, PCND-accumulated cells were exposed to ultrasound and its cytotoxic effects were visualised. The intracellular vaporisation of 9E5-conjugated PCNDs in DLD1 cells was observed by a high-speed imaging system recording 101 subsequent frames at 1000000 frames per second (fps). Figure 3 shows a typical example of intracellular vaporisation processes. Upon exposure to 100 cycles at 4 MHz of ultrasound at a peak negative pressure of 1.5 MPa, the droplets vaporised and cell membranes were ruptured or broken into several parts during this initial stage of vaporisation (Supplementary Movies 3 and 4). Finally, the vaporised PCNDs gushed out of the cells, rupturing the cell membranes. High-speed images clearly show that intracellular vaporisation caused a significant disturbance in cell morphology and destroyed the cells. These results are the first direct evidence that ultrasound exposure of cells after PCND uptake dynamically destroys cells by intracellular vaporisation.

### Cytotoxic efficacy of vaporised 9E5-conjugated PCND

**Figure 4:**
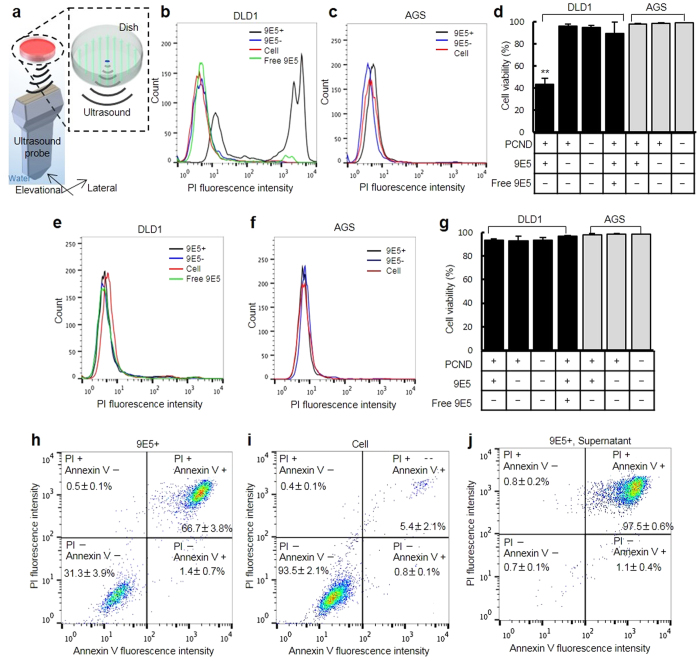
Cytotoxic efficacy of vaporised 9E5-conjugated PCND. (**a**) Schematic diagram of the scanning system used for ultrasound exposure onto 35-mm culture dishes. (**b**–**g**) Flow cytometry assay of the viability of PCND-treated cells in response to ultrasound exposure: (**b**–**d**) ultrasound exposure and (**e**–**g**) unexposure after PI staining. Histograms of the PI fluorescence intensity were obtained by analysing treated DLD1 (**b**,**e**) and AGS cells (**c**,**f**). Cells were treated as described in the legend of Fig. 2c,d. (**d**,**g**) Cell viability was determined as the ratio of PI-fluorescence-negative cells from the histograms. Each bars represent the mean ± S.D. (*N* = 5). **Indicates *p* < 0.01 by one-way ANOVA, followed by the Tukey method. (**h**–**j**) Flow cytometry analysis of apoptotic and necrotic cells by multi-staining with PI and Annexin V. DLD1 cells treated with (**h**) and without 9E5-conjugated PCNDs (**i**) were exposed with ultrasound and then enzymatically harvested from the dishes. (**j**) After ultrasound exposure, cells detached during exposure were collected from the medium before enzymatic harvesting. The PI-negative and Annexin V-positive cells and double-positive cells were identified as apoptotic and necrotic cells, respectively.

We showed that ultrasound treatment destroys cancer cells that have accumulated 9E5-conjugated PCNDs. Next, we quantitatively evaluated the cytotoxic capabilities of vaporised PCNDs using the ultrasound exposure system shown in Fig. 4a. Cell viability was measured by flow cytometry. Five cycles at 5 MHz of pulsed ultrasound with a peak negative pressure of 4.6 MPa was applied using an ultrasound imaging probe. Figure 4b–d shows the fraction of viable cells after ultrasound exposure (*N* = 5). Cell viability was significantly reduced to 43.0 ± 5.6% for 9E5-conjugated PCND-treated DLD1 cells, while there was no significant cell viability decrease for PCNDs without 9E5 conjugation and without ultrasound exposure (Fig. 4b–g). Furthermore, the viability of AGS cells did not decrease. These data indicate that PCND conjugated with 9E5 can sufficiently kill DLD1 cells with high selectivity. The addition of free 9E5 to DLD1 cells before treating/co-treating with 9E5-conjugated PCNDs significantly increased the number of PI− unstained cells (89.5 ± 10.2%). This result is consistent with the decrease in the number of 9E5-conjugated PCNDs taken up by DLD1 cells (Fig. 2e). It is apparent that overexpression of EREG on the target cell surface and 9E5 are essential for the PCND targeting therapy. It is noteworthy that a conventional low-energy ultrasound imaging probe could be used for such treatment.

The 9E5-conjugated PCNDs attached and internalised 97.8 ± 0.5% of DLD1 cells. However, it should be noted that 43.0 ± 5.6% of the ultrasound-exposed cells were viable. In the aforementioned experiments that evaluate the cytotoxic efficacy of intracellular vaporisation, PI was added immediately after ultrasound sonication because we considered that intracellular vaporisation would cause significant cellular morphological changes and immediate cellular death. However, it is possible that some cells endure intracellular vaporisation and then undergo apoptosis. Thus, the proportion of apoptotic cells was measured by Annexin V and PI dual-staining. Figure 4h,i shows the fraction of apoptotic (PI−; Annexin V+) and necrotic cells (PI+) after ultrasound exposure to DLD1 cells treated with or without 9E5-conjugated PCND (*N* = 3). It is apparent that intracellular vaporisation causes necrosis (66.7 ± 3.8%) and not apoptosis (1.4 ± 3.8%). Furthermore, as shown in Fig. 4j, flow cytometry analysis of the supernatant medium showed that most ruptured cells were dead (viability = 0.7 ± 0.1%; *N* = 3).

### Intracellular content release by intracellular vaporisation

**Figure 5:**
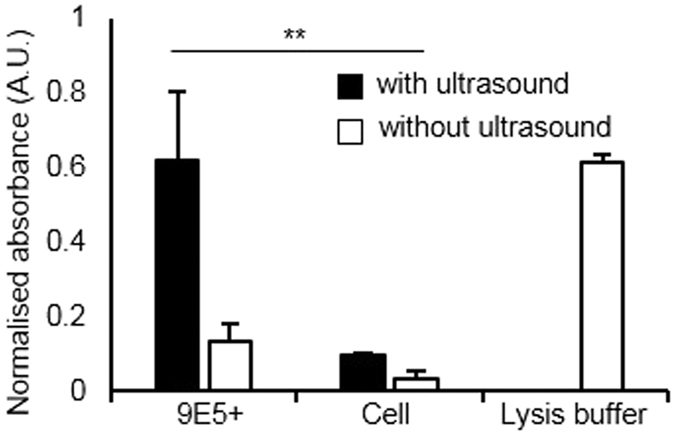
Quantitative evaluation of the release of intracellular components. Extracellular LDH activity was measured using the Cytotoxicity LDH Assay Kit. LDH-mediated formation of formazan dye was quantified by measuring the absorbance at 490 nm using a spectrophotometer. Each bars represent the mean ± S.D. (*N* = 5). **Indicates *p* < 0.01 by one-way ANOVA, followed by the Tukey method).

We showed the selective cytotoxic capabilities of vaporised PCNDs. Furthermore, our high-speed images ascertained that the treated cells release their contents into the extracellular space by intracellular vaporisation, which could potentially provoke an inflammatory response. Intracellular content release was determined using the Cytotoxicity LDH Assay Kit-WST (Dojindo Laboratories, JP) that produces an orange formazan dye from extracellular lactate dehydrogenase (LDH), a cytoplasmic enzyme present inside the cell. The presence of formazan dye was confirmed by measuring the absorbance at 490 nm using a spectrophotometer (NanoDrop 1000 Spectrophotometer, Thermo Fisher, DE, USA). Figure 5 shows the normalised absorbance at 490 nm after exposing PCND-treated cells to ultrasound (black) and in cells without ultrasound (white). Results indicated that the absorbance at 490 nm, which is proportional to the extracellular LDH activity, of 9E5-conjugated PCND-treated DLD1 cells after ultrasound exposure (0.62 ± 0.19) is at the same level as that of cells treated with lysis buffer (0.61 ± 0.02) and 20-fold higher than that of cells without any treatment (0.03 ± 0.02). Thus, intracellular vaporisation can increase their internal contents into the extracellular space.

## Discussion

Antibody-conjugated PCND is a new type of nanomedicine for ultrasound cancer therapy that could be used to treat cancer mechanically by ultrasound with high selectivity. In the present study, 9E5 was conjugated to PCNDs to allow for active targeting and internalisation into DLD1 cells. Both CLSM observations and flow cytometry analysis (Fig. 2) showed the selective targeting capability of 9E5-conjugated PCNDs. Without 9E5 conjugation to PCNDs, neither cell surface attachment nor endocytosis of PCNDs were observed. We achieved excellent targeting capability by conjugation of 9E5 alone, without any external force assistance.

Few studies have reported the conjugation of active targeting molecules to PFC droplets such as aptamer^32^, folate^33^,^34^ and anti-vascular endothelial growth factor receptor 2 antibody with magnetism-assisted targeting^35^ in order to direct them to cancer cells. To obtain cytotoxicity with droplet vaporisation, previous studies have combined anti-cancer drugs such as doxorubicin with droplets^32^,^34^. Marshalek *et al*.^36^ recently demonstrated intracellular delivery and ultrasound activation of intracellular located droplets by decorating folate to the anti-cancer drug-free droplets. However, a cytotoxic effect could not be observed in their study. Ninomiya *et al*.^37^ investigated the targeting ability and cytotoxic effect of anti-cancer drug-free liposomes containing nano-emulsions of perfluoropentane. They modified liposomes with avidin as a targeting ligand for cancer cells and the envelope of hemagglutinating virus of Japan to promote the fusion of liposomes to cells. However, they did not confirm the virus-mediated internalisation of liposomes containing perfluoropentane nano-emulsions toward cancer cells. Moreover, cytotoxic mechanisms were not studied.

Our high-speed imaging shows direct evidence that ultrasound treatment of cells with PCND uptake ruptures the cell membranes by intracellular vaporisation (Fig. 3). Although the location of PCNDs cannot be determined by high-speed imaging, we have confirmed the intracellular location of 9E5-conjugated PCNDs by CLSM observations. Thus, we confirmed that the recorded images are of intracellular vaporisation. This drastic cellular morphological alternation occurred during the initial stages of vaporisation. Therefore, length of the ultrasound duration (number of cycles) would have a less effect on the high-speed imaging results. However, the effect of intracellular vaporisation on surrounding cells would be influenced by the ultrasound parameters because of the alternation in the maximum size of the generated bubbles and oscillation and movement of the bubbles.

Investigations of the selectivity of the cytotoxic effects (Fig. 4) indicate that the decrease in cell viability generated by 9E5-conjugated PCNDs is selective. Ultrasound exposure to the targeted 9E5-conjugated PCNDs significantly decreased the viability of DLD1 cells to 43.0 ± 5.6%. Conversely, PCNDs without 9E5 did not decrease cell viability. Additionally, the cytotoxic effects of 9E5-conjugated PCNDs were not observed in AGS cells, in which EREG expression is relatively low compared to DLD1 cells. To the best of our knowledge, this is the first method reported to mechanically increase cytotoxicity against cancer cells in a highly controllable manner.

One issue that still remains is that the fraction of DLD1 cells targeted by 9E5-conjugated PCND (Fig. 2e) and cell viability after ultrasound exposure (Fig. 4d) were inconsistent. A possible reason is that some portion of intracellular PCNDs remained inert and did not cause a biological effect under the ultrasound exposure conditions. Most cells, in which the vaporisation of PCND occurred, were detached from the dish surface after ultrasound exposure to 9E5-conjugated PCND. Cells that would be included in the supernatant were mostly the necrotic cells, as shown in Fig. 4j (viability = 0.7 ± 0.1%; *N *= 3). Therefore, we assumed that once vaporisation was achieved, cells could undergo necrosis. Hence, further improvement or optimisation of exposure conditions is required to kill all PCND-incorporated cells, and this will be done in our future research.

We proposed the use of an antibody as a cancer cell-targeting tool in combination with PCND-vaporisation as a nanomedicine for ultrasound cancer therapy. 9E5-conjugated PCNDs have several benefits for cancer treatment owing to their active targeting functionality. Treatment strategies for the proposed ultrasound cancer therapy will not require precise identification of the treatment area owing to the selective adsorption and accumulation of 9E5-conjugated PCNDs inside the target cells. Furthermore, the therapeutic effects are exerted by physical actions, thus avoiding concerns about drug resistance and biological variability between cancer types. This approach can be potentially used to damage any type of cancer cell by selecting the appropriate antibody.

Beyond its use as a dynamic therapeutic agent, it could potentially activate the human-inherent immune system to further aid in the death of cancer cells^38^,^39^. Our high-speed images and extracellular LDH activity analysis ascertain that the treated cells release their contents into the extracellular space by intracellular vaporisation, which could potentially provoke an inflammatory response^40^. This acute inflammation caused by vaporisation-induced necrosis might improve cancer immunity through antigen presentation^41^. Therefore, 9E5-conjugated PCNDs could be the next generation of both ultrasound-activated dynamic nanomedicine and cancer immunotherapy. The effects of intracellular vaporisation on the immune system must be considered in future studies. It is noteworthy that the proposed ultrasound cancer therapy can be conducted using a conventional ultrasound imaging probe with low energy, as this factor will promote easy clinical translation. Furthermore, it has the potential to treat cancer cells without affecting adjacent normal cells. As shown in Fig. 2a and Supplementary Fig. 3, PCNDs are transported inside cells in a short period of time. The ultrasound-induced vaporisation of intracellular PCNDs allows for high selectivity in cell killing. Hence, it would beneficial for treating cancers such as glioblastoma, in which important tissues are involved. It also might be effective for treating cancers such as peritoneal metastasis and hepatoma, where scattered micro-masses make the conservation of vital organs difficult.

## Methods

### Preparation of Biotinylated 9E5 antibody

We chose anti-epiregulin antibody (9E5) as the active targeting agent, with the potential to induce internalisation of PCND. 9E5 hybridoma cells were intraperitoneally implanted in BALB/c nude mice, and ascites were obtained and purified on a Protein G column. The 9E5 monoclonal antibody was generated as previously described^26^,^42^. All animals were maintained in accordance with the regulations set by The University of Tokyo, and all animal experiments were conducted following the institutional guidelines. Animal study protocol was approved by The University of Tokyo (#RAC120101). A solution containing 9E5 (250 μL) was dialysed against 1 L of 100 mM boric buffer (pH 8.3) at 4 °C overnight, and the concentration of 9E5 in the dialysed solution was estimated by measuring absorbance at 280 nm. A molar extinction coefficient of 220000 M^−1 ^cm^−1^ was used to calculate the concentration of 9E5. One mM of Sulfo-NHS-activated biotin derivatives (EZ-Link Sulfo-NHS-LC-Biotin, Life Technologies, Carlsbad, CA, USA) in dry DMSO, freshly prepared right before the experiment, was then added to the antibody solution at a final concentration of 5 eq. of 9E5 to conduct biotinylation of the antibody. The reaction took place overnight at 4 °C. To quench the remaining activated biotinylated compound, 100 μL of 100 mM Tris–acetate (pH 7.0) was added and allowed to react for 1 hour at 4 °C. Finally, biotinylated 9E5 was concentrated by centrifugation using an ultra-membrane filter of 100 kDa MWCO (Amicon Ultra 0.5 mL, Millipore, US); the buffer was exchanged to PBS. The concentration of biotinylated 9E5 was measured as described above.

### Preparation of AF647-labelled 9E5-conjugated PCND

The biotinylated-PCNDs (internal composition, (1:1) mixture of perfluoropentane and perfluorohexane) were prepared as described elsewhere^43^,^44^ and provided by Central Research Laboratory Hitachi (Tokyo, JAPAN). The mixture composition ratio of 1:1 was chosen to ensure thermal stability (boiling point calculated to be 40 °C) and sensitivity to activation by ultrasound^44^. Biotinylated-9E5 antibody and Alexa Fluor 647 conjugated streptavidin (SA-AF647) (Life Technologies, US) were mixed in PBS at concentrations of 0.4 μM each in a total volume of 50 μL and incubated for 15 min at room temperature to form AF647-labelled 9E5-SA conjugates. The biotinylated PCND dispersion was diluted 20-fold with cold PBS and mixed with the solution containing AF647-labelled 9E5-SA conjugate at a volume ratio of 1:1. The mixture was then incubated for 30 min on ice. The unconjugated 9E5 and SA-AF647 were removed by centrifugation at 3000 × g for 5 min and the supernatant was discarded. The precipitated AF647-labelled 9E5-conjugated PCNDs were dispersed in 100 μL of cold PBS containing 20% glycerol and centrifuged again under the same conditions as described above to wash PCNDs. This washing step was repeated twice. Finally, the AF647-labelled 9E5-conjugated PCNDs were dispersed in 1 mL of cold RPMI and kept on ice until use. The particle-size distribution of the AF647-labelled 9E5-conjugated PCND suspensions was measured using a laser diffraction particle analyser (LS13320, Beckman Coulter, US). For negative control experiments, non-9E5-conjugated (SA-biotin conjugated) AF647-labelled PCNDs were prepared using biotin instead of biotinylated-9E5 as described above.

### Cell culture

DLD1 and AGS cells were cultured in 35-mm cell culture dishes at an initial density of 2 × 10^5^ cells/dish with RPMI medium supplemented with 10% fetal bovine serum and incubated at 37 °C in a humidified atmosphere with 5% CO_2_. All the experiments were conducted one day after seeding on 35-mm culture dishes. The same procedure was used when cells were cultured in the 35-mm glass-bottomed dishes.

### Targeting ability: CLSM and flow cytometry analysis

DLD1 and AGS cells were cultured in 35-mm glass-bottomed dishes as described above. Intracellular delivery of 9E5-conjugated PCNDs into DLD1 cells was verified using CLSM (LSM510 META-ConfoCor 3, Carl Zeiss, DE). The use of AF647 probes permits the detection of internalised 9E5-conjugated PCNDs. After a 24-hour incubation, all the culture medium in the dish was aspirated, the cells were washed with PBS and 1 mL of RPMI-diluted 9E5-conjugated PCNDs solution was added to the cells. After a 3-hour incubation with/without 9E5-conjugated PCNDs, all the solutions were removed. Approximately 10 mL of RPMI was added to the dish to fill it with the media. A dish cap was placed on top, and the dish was inverted so that non-attached PCNDs would settle out, since the density of PCND (1.6 g/mL) is higher than that of the media. This washing procedure was repeated twice; lastly, 2 mL of fresh RPMI was added, and cells were observed by CLSM. For cellular membrane labelling, Vybrant Dio (Invitrogen Corporation, Carlsbad, CA) was used (see Supplementary Section ‘Cell membrane labelling’).

Additionally, the targeting capability of AF647-labelled 9E5-conjugated PCNDs to DLD1 cells was quantitatively measured by flow cytometry (BD FACSCalibur, BD Biosciences, SanJose, CA, USA). DLD1 and AGS cells were cultured in 35-mm cell culture dishes (2 × 10^5^ cells/dish). The AF647-labelled 9E5-conjugated PCNDs were introduced to the DLD1 cells in the same manner as for the CLSM observation and analysed by flow cytometer (see Supplementary Section ‘Flow cytometry’).

As additional negative controls for the experiments, the cells with non-9E5-conjugated AF647-labelled PCNDs and those without PCNDs (cells alone) were incubated in the same manner for both CLSM observation and flow-cytometry analysis. Furthermore, we considered that AF647-labelled 9E5-conjugated PCND uptake might be inhibited by the addition of free 9E5 to DLD1 cells, free 9E5 should bind to EREG and block the binding of AF647-labelled 9E5-conjugated PCNDs. Hence, 1 mL of 0.5 μM 9E5 antibody solution was added to the DLD1 cells and incubated for 30 min before adding AF647-labelled 9E5-conjugated PCNDs.

### High-speed imaging of intracellular vaporisation

Intracellular vaporisation of 9E5-conjugated PCNDs was observed using the high-speed imaging system shown in Fig. 3. A custom-made, nose-cone-shaped wave guide^45^ mounted 4-MHz single element piezoelectric transducer with a focal length of 25 mm (17.5 × 7.9 mm, Type C213, Fuji Ceramics, JP) was developed to enable sonication to DLD1 cells during high-speed imaging. The transducer was connected to a multi-function generator (WF1974, NF Corporation, JP) and a 50-dB broadband amplifier (2100 L, Electronics & Innovation, US). The wave number was set to 100 cycles at 4 MHz (pulse duration, 25 μs). The waveguide was filled with ultrasonic gel, and the nose was fully covered with a paraffin film to ensure ultrasound propagation through the waveguide. DLD1 cells were exposed to a peak negative pressure of 1.5 MPa. The multi-function generator was also connected to a high-speed imaging camera (Hyper Vision HPV-1, Shimadzu, JP) coupled with an inverted microscope (Eclipse Ti-U, Nikon, JP) equipped with an oil immersion 60× objective lens to synchronise the sonication and the recording of images by high-speed camera. A super high-pressure mercury lamp (C-LHG1, Nikon, JP) connected to a power supplier (C-SHG1, Nikon, JP) was used for the illumination light source. The high-speed camera was set to a frame rate of 0.25, 1Mfps and an exposure time of 0.5 μs. A total of 101 subsequent frames was recorded, and the image resolution was 312 × 260 pixels. The 9E5-conjugated PCND-targeted DLD1 cells were prepared in glass-bottom dishes and placed on top of the objective lens. The transducer was positioned by a three-axis motorised stage, and the angle was set to 45° with a custom-made holder. PBS was added inside the dish until the tip of the waveguide was fully covered.

### Flow cytometry analysis of cytotoxic efficacy

The cytotoxic efficacy of vaporisation of 9E5-conjugated PCNDs was investigated in cultured DLD1 and AGS cells using the experimental system shown in Fig. 4a. An ultrasound imaging probe (EUP-L73S, Hitachi Aloka Medical, JP) connected to a programmable ultrasound imaging system (V1, Verasonics, Kirkland, WA, USA) was used for delivering vaporisation pulses to the PCNDs. The cell culture dishes were positioned 36 mm from the transmission surface of the ultrasound imaging probe with a custom-made dish holder connected to a single axis motorised stage (ALZ-115-E1P, Chuo Precision Industrial, JP). The pulse length was set to 5 cycles at 5 MHz with a peak negative pressure of 4.6 MPa (measured at the bottom of the dish), and the focus of the transmitted ultrasound was set at 38 mm from the transmission surface of the ultrasound imaging probe. Such short pulses were used to avoid unintended cavitation that damages cells non-specifically. The acoustic profile measured at the bottom of the dish is shown in Supplementary Fig. 4. The focus was electronically scanned from −1.20 mm to 1.20 mm (centre of the probe was set to 0 mm) with a 0.20-mm pitch in the lateral direction of the probe; the pulse repetition frequency was set to 0.5 kHz. These sonication conditions are known to achieve successful vaporisation of PCNDs^31^. The probe was positioned by a two-axis motorised stage (ALD-604-E1P, Chuo Precision Industrial, JP) with a 0.30-mm pitch in the elevational direction and 1.20-mm pitch in the lateral direction, both at an interval of 100 ms. Trigger signals were transmitted to the programmable ultrasound imaging system at each position to expose 6 sets of the above-mentioned vaporisation pulses. A water tank was filled with degassed water, and the temperature was maintained at 37 °C.

DLD1 and AGS cells were cultured in 35-mm cell culture dishes at a density of 2 × 10^5^ cells/dish. The 9E5-conjugated PCNDs were introduced to the cells in the same manner as described for the targeting capability measurements. As a negative control for the experiments, cells were incubated with non−9E5-conjugated AF647-labelled PCNDs and without PCNDs. All the samples were tested with and without ultrasound sonication. Furthermore, for DLD1 cells treated with AF647-labelled 9E5-conjugated PCNDs, EREG blocking was conducted in the same manner as described above. PI (25535-16-4, Dojindo Laboratories, JP) was added to the dishes just after the sonication, resulting in a final concentration of 1 μL/mL. Cell viabilities were quantitatively measured by flow cytometry (see Supplementary Section ‘Flow cytometry’). PI is used to distinguish between viable and non-viable cells.

The proportion of apoptotic cells was measured by Annexin V and PI dual-staining using the Annexin V-FITC Apoptosis Detection Kit (BioVision Incorporated, CA, USA). DLD1 cells treated with or without 9E5-conjugated PCNDs were exposed to ultrasound. The supernatant was collected in a tube after ultrasound exposure. To remove adhered cells, 1 mL of PBS was added to the dish and added to the same tube containing the supernatant and 100 μL 0.25% EDTA–trypsin solution was added to the dishes. After 3 min of incubation, 2 mL of PBS was added, and all the medium was collected in the same tube. The tube was centrifuged at 1400 rpm for 3 min, and the supernatant was aspirated. The pellet was resuspended in 500 μL of binding buffer (BioVision Incorporated, CA, USA), and 5 μL of Annexin V-FITC and PI was added. After 5 min of incubation at room temperature, the samples were kept on ice until just before the measurement. For supernatant viability measurement, trypsinisations were excluded.

### Intracellular content release: LDH activity analysis

Intracellular content release was determined using the Cytotoxicity LDH Assay Kit-WST (Dojindo Laboratories, JP). Extracellular LDH activity was quantified using a spectrophotometer (NanoDrop 1000 Spectrophotometer, Thermo Fisher, DE, USA) by measuring the absorbance of formazan dye at 490 nm. DLD1 cells were cultured in 35-mm cell culture dishes at a density of 2 × 10^5^ cells/dish. The 9E5-conjugated PCNDs were introduced to the cells in the same manner as that described for the targeting capability measurements, and the cells were exposed to ultrasound in the same manner as that described for the cytotoxicity measurements. The supernatant was collected in a tube after ultrasound exposure. The tube was centrifuged at 1400 rpm for 3 min, and 100 μL of supernatant was mixed with 100 μL of the assay medium. After 30 min of incubation at room temperature without light exposure, 50 μL of stop solution was added and the absorbance at 490 nm was measured using the spectrophotometer. As additional negative controls for the experiments, the cells without PCNDs (cells alone) were incubated in the same manner for both CLSM observation and flow cytometry analysis. All the samples were tested with or without ultrasound sonication. As additional positive controls for the experiments, the cells were treated with lysis buffer (Dojindo Laboratories, JP). All the culture medium in the dish was aspirated, the cells were washed with RPMI, and 2 mL of RPMI-diluted lysis buffer (RPMI = 1.8 mL; lysis buffer = 0.2 mL) was added to the cells. After 30 min of incubation at 37 °C in a humidified atmosphere with 5% CO_2_, an assay medium was added in the same manner as that described above.

### Statistical analysis

Analysis of variance (ANOVA) was used to establish the significance between the different experimental groups. The Tukey method was applied to evaluate the significance of differences between the groups.

## Additional Information

**How to cite this article:** Ishijima, A. *et al*. Selective intracellular vaporisation of antibody-conjugated phase-change nano-droplets *in vitro. Sci. Rep.* **7**, 44077; doi: 10.1038/srep44077 (2017).

**Publisher's note:** Springer Nature remains neutral with regard to jurisdictional claims in published maps and institutional affiliations.

## Supplementary Material

Supplementary Movie 1

Supplementary Movie 2

Supplementary Movie 3

Supplementary Movie 4

Supplementary Information
