# Supplementary material for: Selective intracellular vaporisation of antibody-conjugated phase-change nano-droplets in vitro
Source: Sci Rep. 2017 Mar 23;7:44077. doi: 10.1038/srep44077 (PMC5363066; doi:10.1038/srep44077)
Supplement: Supplementary Information [file srep44077-s5.doc]

**Supplementary Information**

**Selective intracellular vaporisation of antibody-conjugated phase-change nano-droplets *in vitro***

A.Ishijima^1,†^, K.Minamihata^2,3,†^, S.Yamaguchi^4,†^, S.Yamahira^3^, R.Ichikawa^1^, E.Kobayashi^1^,

M.Iijima^4^, Y.Shibasaki^4^, T.Azuma^5,*^, T.Nagamune^3,6,*^& I. Sakuma^1,6,7,*^

^1^Department of Precision Engineering, School of Engineering, The University of Tokyo, Tokyo 113-8656, Japan.

^2^Department of Applied Chemistry, Kyushu University, Fukuoka 819-0395, Japan.

^3^Department of Chemistry & Biotechnology, School of Engineering, The University of Tokyo, Tokyo 113-8656, Japan.

^4^Research Center for Advanced Science and Technology, The University of Tokyo, Tokyo 153-8904, Japan.

^5^Center for Disease Biology and Integrative Medicine, The University of Tokyo, Tokyo 113-8656, Japan.

^6^Department of Bioengineering, The University of Tokyo, Tokyo 113-8656, Japan.

^7^Medical Device Development and Regulation Research Center, School of Engineering, The University of Tokyo, Tokyo 113-8656, Japan.

^†^These authors contributed equally to this work.

^*^Corresponding authors

I. Sakuma E-mail address: sakuma@bmpe.t.u-tokyo.ac.jp

T. Nagamune E-mail address: nagamune@bioeng.t.u-tokyo.ac.jp

T. Azuma E-mail address: azuma@fel.t.u-tokyo.ac.jp

**CLSM observation of fluorescent-labelled 9E5 antibody in living cells**

To visualise 9E5 antibody accumulation on and internalisation in high-EREG-expressing cells, DLD1 cells were observed in a culture medium including fluorescent-labelled 9E5 using CLSM. First, Alexa Fluor 647 (AF647)-labelled 9E5 was prepared by incubating 9E5 with AF647 *N*-hydroxysuccinimidyl ester (AF647-NHS) (Invitrogen Corporation, US) in borate buffer (100 mM, pH 8.3) for 2 h. The final concentrations of 9E5 and AF647-NHS in the reaction mixture were 12.3 and 123 µM, respectively. After incubation, the labelled 9E5 was purified by dialysis in PBS, and the concentration of the labelled 9E5 solution was determined from the absorbance of light at wavelengths of 280 and 647 nm using the molar extinction coefficients of antibody (*ε*_280_: 22000) and AF647 (*ε*_647_: 239000, *ε*_280_/*ε*_647_: 0.030). Next, DLD1 cells were cultured on a 35-mm glass-bottom dish in a culture medium of RPMI 1640 supplemented with 10% FBS for 1 day. After removing the medium, the culture medium including 50 µM AF647-labelled 9E5 was added in a dish on ice, and DLD1 cells were then observed using CLSM under 5% CO_2_ at 37°C. To obtain time-course images of the cells under treatment with AF647-labelled 9E5, DLD1 cells were successively cultured under observation on the microscope stage. After culture for various periods under 5% CO_2_ at 37°C, the CLSM images of the same area were obtained.

**Cell membrane labelling**

For cellular membrane labelling, Vybrant Dio (Invitrogen Corporation, US) was used. After the washing procedure described in the manuscript, cells were washed with 1 mL of 1% BSA/PBS and 200 μL of 5 μM DiO solution diluted with serum-free RPMI was added to the dish and incubated for 3 min. In total, 1 mL of 1% BSA/PBS was added, and the medium was aspirated; this procedure was repeated twice. Serum-free RPMI (2mL) was added to the dish and observed by CLSM.

**Flow cytometry**

The targeting capability and therapeutic efficacy of the 9E5-conjugated PCNDs to DLD1 cells were quantitatively measured by flow cytometry (BD FACSCalibur, BD Biosciences, US). DLD1 and AGS cells were cultured in 35-mm cell culture dishes (2 × 10^5^ cells/dish). The 9E5-conjugated PCNDs were introduced to the DLD1 cells in the same manner as for CLSM observation. To measure the targeting capability, all the media was removed to a tube after the 3-hour incubation. For therapeutic efficacy measurement, all the media was removed to a tube after ultrasound exposure and PI addition. To remove adhered cells, 1 mL of PBS was added to the dish and removed in the same tube and 100 μL 0.25% EDTA-trypsin solution added to the dishes. After 3 min of incubation, 2 mL of PBS was added, and all the media was removed to the same tube. The tube was centrifuged at 1400 rpm for 3 min and the supernatant was aspirated. The pellet was resuspended in 200 µL of cold PBS and kept on ice until just before the measurements.

| 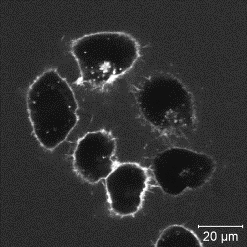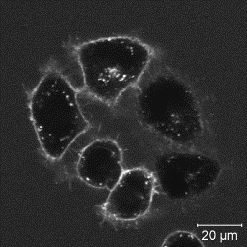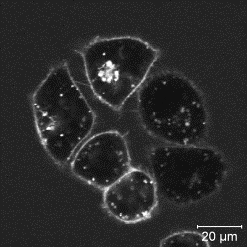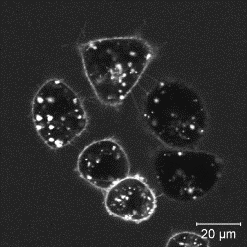 0h  1h  2h  4h |
| --- |
| **Supplementary Figure 1. CLSM images of cells targeted with 9E5 antibody.** CLSM observation of DLD1 cells after treatment with fluorescent-labelled 9E5 for various periods from 0 to 4 h. Fluorescent images of Alexa Fluor 647 (AF647) were successively obtained from living DLD1 cells in culture media including 50 μM AF647-labelled 9E5 at 37°C. |

|  |
| --- |
| **Supplementary Figure 2. CLSM images of cells targeted with PCNDs.** CLSM observation of 9E5-mediated selective accumulation and internalisation of 9E5-conjugated PCNDs to DLD1 cells (9E5^+^, 9E5-conjugated PCNDs; 9E5^−^, non-9E5-conjugated-PCNDs; Cell, without PCNDs; DIC, differential interference contrast image; AF647, pink fluorescent image of Alexa Fluor 647, which was labelled to SA; DiO, green fluorescent image of DiO). These images were obtained after treatment of AF647-labelled 9E5-conjugated PCNDs and staining with DiO. Scale bars were 50 μm (see also Supplementary Movies 1 and 2). |

| 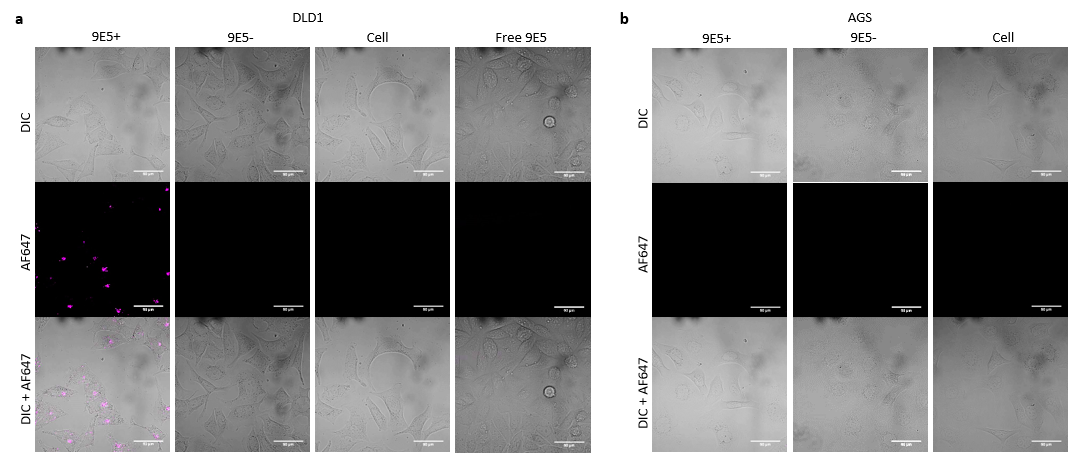 |
| --- |
| **Supplementary Figure 3. CLSM images of cells targeted with PCNDs.** (**a**) CLSM observation of 9E5-antibody-mediated selective accumulation and internalisation of 9E5-conjugated PCNDs to DLD1 cells. (9E5^+^, 9E5-conjugated PCNDs; 9E5^−^, non-9E5-conjugated-PCNDs; Cell, without PCNDs; DIC, differential interference contrast image; AF647, pink fluorescent image of Alexa Fluor 647, which was labelled to SA; Merge, merging of AF647 and DIC images). (**b**) The same observations of AGS cells. Scale bars were 50 μm. |

| 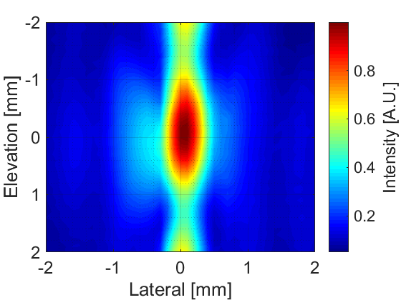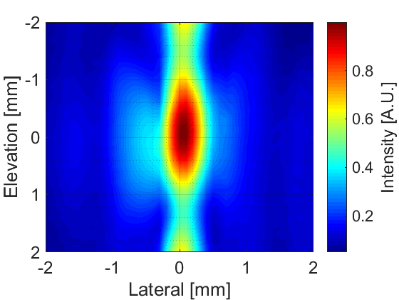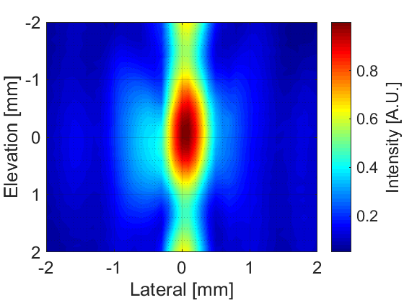 |
| --- |
| **Supplementary Figure 4. Acoustic profile of ultrasound imaging probe.** The acoustic profile was measured at the bottom of the dish by a hydrophone. |

**Supplementary Movie 1. Stack image sequences of membrane-stained DLD1 cells treated with 9E5-conjugated PCNDs (pink, 9E5-conjugated PCNDs; green, cell membrane).**

**Supplementary Movie 2. 3-D projected stack image sequences of membrane-stained DLD1 cells treated with 9E5-conjugated PCNDs (pink: 9E5-conjugated PCNDs; green, cell membrane).**

**Supplementary Movie 3. Intracellular vaporisation of internalised 9E5-conjugated PCNDs taken at 0.25 Mfps.**

**Supplementary Movie 4. Intracellular vaporisation of internalised 9E5-conjugated PCNDs taken at 1 Mfps.**
